# Fecal Calprotectin: A Reliable Predictor of Mucosal Healing after Treatment for Active Ulcerative Colitis

**DOI:** 10.1155/2017/2098293

**Published:** 2017-10-31

**Authors:** Vendel Kristensen, Arne Røseth, Tahir Ahmad, Viggo Skar, Bjørn Moum

**Affiliations:** ^1^Unger-Vetlesen's Institute, Lovisenberg Diaconal Hospital, Oslo, Norway; ^2^Department of Gastroenterology, Oslo University Hospital, Oslo, Norway; ^3^Department of Medicine, Lovisenberg Diaconal Hospital, Oslo, Norway; ^4^Institute of Clinical Medicine, University of Oslo, Oslo, Norway

## Abstract

**Objectives:**

Mucosal healing has become the new goal of treatment in ulcerative colitis. Fecal calprotectin has been demonstrated to differentiate between mucosal inflammation and mucosal healing. With this project, we investigated whether a reduction in f-calprotectin to <250 *μ*g/g after medical treatment for active ulcerative colitis could predict mucosal healing.

**Material and Methods:**

After a baseline colonoscopy, 20 patients with active ulcerative colitis were followed with consecutive fecal calprotectin monthly until two measurements of fecal calprotectin < 250 *μ*g/g or a maximum follow-up of 12 months. A flexible sigmoidoscopy was then performed and Mayo endoscopic subscore was used to evaluate degree of inflammation. Simple Clinical Colitis Activity Index was used for evaluation of clinical disease activity.

**Results:**

A total of 16 patients achieved fecal calprotectin < 250 *μ*g/g during follow-up, and all 16 patients had endoscopic mucosal healing (Mayo endoscopic subscore of ≤1) on the second endoscopy. The remaining four patients had persistently high f-calprotectin levels before the second endoscopy with Mayo endoscopic subscore corresponding to endoscopic mucosal healing in three out of four patients.

**Conclusions:**

Fecal calprotectin <250 *μ*g/g after medical treatment for active ulcerative colitis is a reliable marker of endoscopic mucosal healing.

## 1. Introduction

Ulcerative colitis (UC) is a chronic disease characterized by intestinal inflammation. The clinical course is typical with periods of remission interrupted by episodes of relapse. Traditionally, treatment of UC aimed to reduce the inflammation during relapse and maintain control of symptoms. Endoscopic remission reduces the risk of clinical relapse, hospitalization, and future colectomy in UC [1–3], leading to mucosal healing as the new goal of treatment in recent years. Fecal- (f-) calprotectin is demonstrated to correlate well with endoscopic disease activity, in contrast to clinical activity indices and inflammatory markers such as C-reactive protein, erythrocyte sedimentation rate, and leukocytes [4–6]. Consecutive f-calprotectin measurements to evaluate response to medical treatment in clinical trials and during clinical follow-up represent an interesting noninvasive strategy, but require further studies.

A recent study evaluated the effect of infliximab induction therapy in patients with UC using weekly f-calprotectin measurements for ten weeks and found f-calprotectin less than 50 *μ*g/g to correlate excellently with endoscopic remission defined as Mayo endoscopic subscore ≤ 1 [7]. The optimal f-calprotectin cut-off level separating endoscopic active UC from UC in endoscopic remission is though probably higher than 50 *μ*g/g and depends on the assay used for analysis [8, 9].

Our aim in this study was to evaluate if f-calprotectin below a predefined cut-off level after treatment for active UC was a reliable biomarker of endoscopic mucosal healing.

## 2. Materials and Methods

### 2.1. Patients

Between December 2012 and October 2014, adult patients with active UC were included in the study. Both newly diagnosed patients and patients with a flare of established disease were invited to participate.

### 2.2. f-calprotectin

The patients were instructed to provide a feces sample 3 days after the baseline colonoscopy and then monthly during follow-up.

The feces samples were stored at −20°C until analysis by an enzyme-linked immunosorbent assay (ELISA) (Bühlmann Laboratories AG, Schönenbuch, Switzerland) according to the manufacturer's instructions. The analytical range was between 30 and 1800 *μ*g/g. Samples with <30 *μ*g/g were registered as 29 *μ*g/g and >1800 *μ*g/g as 1801 *μ*g/g.

### 2.3. Assessment of Disease Activity

All patients had a colonoscopy performed at inclusion. Montreal classification for disease extent [10] was registered and endoscopic disease activity assessed using the Mayo endoscopic subscore [11]. The segment with the most severe inflammatory activity was chosen to set the score. After two consecutive f-calprotectin measurements < 250 *μ*g/g, or after one year of follow-up without achieving two consecutive f-calprotectin measurements < 250 *μ*g/g, a flexible sigmoidoscopy was performed. A flexible sigmoidoscopy was chosen as the second endoscopy, as this is less invasive than a full colonoscopy. Three days after the second endoscopy, the patients were asked to send another feces sample.

Routine serological markers and Simple Clinical Colitis Activity Index (SCCAI) [12] were registered at the time of the first and second endoscopy.

### 2.4. Statistical Analyses

Data are presented as medians with interquartile ranges (IQR) unless otherwise specified. Clinical performance characteristics are presented as point estimates of the mean with 95% confidence intervals (CI).

Statistical analyses were performed using IBM SPSS Statistics version 22 (SPSS Inc., Chicago, IL).

### 2.5. Ethical Considerations

The study was approved by the Norwegian South East Regional Committee for Medical and Health Research Ethics. All study participants signed an informed consent before entering the study.

## 3. Results

**Figure 1:**
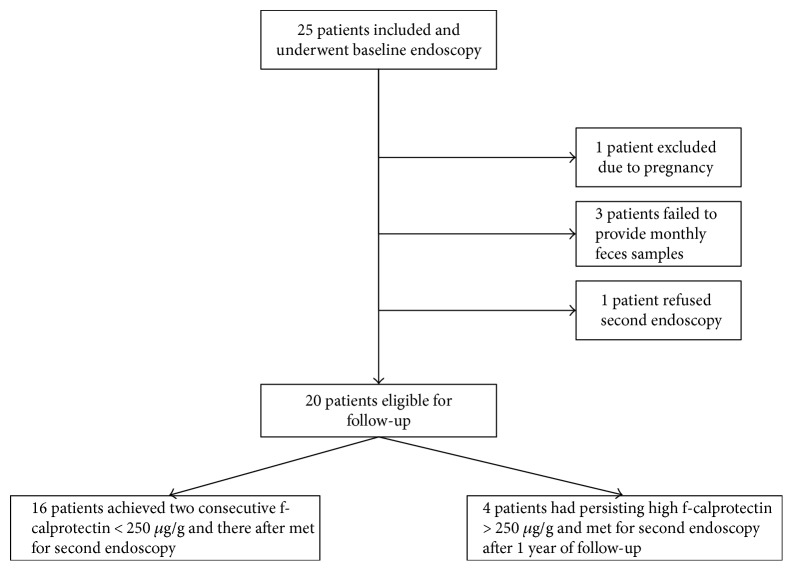
Flow chart of patients included in the study.

**Figure 2:**
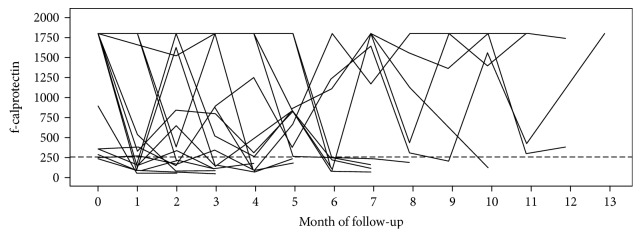
The figure demonstrates how the f-calprotectin levels evolved within each study participant during the study period. Broken line represents the cut-off of 250 *μ*g/g. Within three months, six patients had values below cut-off. Within six months, 13 patients had values below cut-off. Within 10 months, 16 patients had values below cut-off. The remaining four patients never achieved values below cut-off.

Altogether, 25 UC patients were included. During the follow-up, five patients were excluded or lost to follow-up (Figure 1). Baseline characteristics for the 20 patients included in the analysis are presented in Table 1.

f-calprotectin values for all patients during the study period are presented in Figure 2. During follow-up, 16 patients achieved two consecutive f-calprotectin measurements < 250 *μ*g/g. The duration from baseline endoscopy and until f-calprotectin < 250 *μ*g/g was reached, ranging from two to 10 months, with a median of four months. All 16 patients had at the follow-up sigmoidoscopy a Mayo endoscopic subscore of ≤1. Two out of these 16 patients had a SCCAI score of >2.5 at the time of the second endoscopy.

Four patients had persistently elevated f-calprotectin and underwent a flexible sigmoidoscopy one year after the baseline colonoscopy. The results from follow-up of these patients are presented in Table 2.

Table 3 presents the sensitivity and specificity of two consecutive f-calprotectin < 250 *μ*g/g as a surrogate marker of endoscopic mucosal healing.

## 4. Discussion

Two consecutive f-calprotectin <250 *μ*g/g predict endoscopic mucosal healing (Mayo endoscopic subscore of ≤1) with a high positive predictive value, as demonstrated in this study. The negative predictive value was low, with wide confidence intervals, due to a limited sample size. Only one out of four patients with persisting high f-calprotectin had not achieved endoscopic mucosal healing.

UC patients often report coinciding irritable bowel syndrome-like symptoms that might be misinterpreted as relapse or persisting disease activity [13–15]. SCCAI < 2.5 are demonstrated to correlate with patient-defined remission with a sensitivity of 79% and a specificity of 82% [16]. In our study of patients achieving f-calprotectin < 250 *μ*g/g and with endoscopic mucosal healing, there were still two patients with SCCAI > 2.5. This is a challenge in clinical practice. In the European guidelines for endoscopy in inflammatory bowel disease (IBD), f-calprotectin is proposed as a potential surrogate marker of mucosal healing [17]. Our study supports this strategy, as f-calprotectin < 250 *μ*g/g predicts endoscopic mucosal healing with high positive predictive value and may therefore be regarded as a reliable noninvasive biomarker.

As described previously, four out of 20 patients did not achieve two consecutive f-calprotectin values < 250 *μ*g/g after standard medical treatment. All four patients were in clinical remission according to SCCAI. However, one patient still had endoscopic ulcerations corresponding to Mayo endoscopic subscore of 2. Such patients represent another challenge in clinical practice. The goal of treatment should be endoscopic mucosal healing; however, endoscopic monitoring is costly and unpopular amongst patients.

Elevated f-calprotectin is found to precede clinical relapse in clinically quiescent UC [18, 19]. In a study of UC patients in endoscopic remission defined as Mayo endoscopic subscore of 0, low f-calprotectin predicted sustained remission better than absence of histological inflammation in colonic biopsies [20]. F-calprotectin is therefore suggested as a potential target of treatment itself.

A randomised case-control study has demonstrated that elevated f-calprotectin levels can be decreased by intensifying 5-ASA treatment in UC patients [21]. Another study evaluated 5-ASA dose escalation in UC patients monitored with monthly f-calprotectin measurements. UC patients in remission were randomised to a control group or an intervention group. The intervention group received dose escalation based on elevated f-calprotectin only. The authors found a tendency towards decrease of relapses in the intervention group [22]. Patients with active UC receiving medical treatment may also benefit from a similar strategy. f-calprotectin could be monitored and treatment evaluated and potentially intensified according to response.

Two consecutive elevated samples seem to be more accurate than one in predicting a forthcoming flare [23]. Intraindividual day-to-day f-calprotectin variability has been described in patients without colonic inflammation and neoplasms (“patient controls”) and in IBD patients [24–26]. This variation is even described within the same day in patients with active UC [26, 27], which may be confusing in clinical practice. However, we have previously demonstrated that f-calprotectin levels crossed proposed cut-off levels in only a minority of UC patients [28]. Nevertheless, one single f-calprotectin indicative of endoscopic mucosal healing should be confirmed with a consecutive control.

In the presented study, three out of four patients with persisting f-calprotectin ≥ 250 *μ*g/g had Mayo endoscopic subscore of ≤1 on the second endoscopy. These may be patients in risk of forthcoming flare. However, it is important to bear in mind that the endoscopic evaluation after treatment was a flexible sigmoidoscopy. F-calprotectin is a marker of inflammation throughout the entire gastrointestinal tract, and these patients may have had patchy inflammation due to a partial treatment response. A full colonoscopy could have clarified this.

A limitation in our study is the small sample size, hence wide confidence intervals for sensitivity and specificity. Our study was single-center and exploratory, and a future larger multicenter study will therefore be appreciated.

In conclusion, f-calprotectin seems a useful marker of endoscopic mucosal healing after treatment for active UC. Further studies are needed to evaluate the clinical importance of high f-calprotectin in patients in endoscopic remission.

## Figures and Tables

**Table 1:** Baseline characteristics of the study participants.

Number of patients	20
Age, median years (range)	31 (18–60)
Male, *n* (%)	6 (30)
SCCAI, median (IQR)	8 (4)
CRP, median (IQR)	4.0 (12)
Hb, median (IQR)	13.0 (1.2)
Ferritin, median (IQR)	43 (58)
Platelets, median (IQR)	328 (193)
Baseline fecal calprotectin, median *μ*g/g (IQR)	1801 (1453)
Montreal classification, *n* (%)	
Left-sided colitis	10 (50)
Extensive colitis	10 (50)
Mayo endoscopic subscore, *n* (%)	
1	2 (10)
2	9 (45)
3	9 (45)
Medical treatment, *n* (%)	
5-ASA	20 (100)
Prednisolone	1 (5)
Thiopurine	4 (20)
Methotrexate	1 (5)
Anti-TNF	3 (15)

SCCAI: Simple Clinical Colitis Activity Index; IQR: interquartile range; CRP: c-reactive protein; Hb: haemoglobin.

**Table 2:** Results from follow-up of the four patients with persisting fecal calprotectin > 250 *μ*g/g throughout the study period.

		MES	SCCAI	f-calprotectin	
				Last value prior to endoscopy	3 days after endoscopy
Pt 1	Baseline	3	6	NA	348
	One year follow-up	**2**	0	>1800	missing
Pt 2	Baseline	2	4	NA	350
	One year follow-up	**1**	0	373	44
Pt 3	Baseline	1	7	NA	>1800
	One year follow-up	**0**	1	>1800	502
Pt 4	Baseline	3	10	NA	>1800
	One year follow-up	**1**	0	>1800	967

Pt: patient; MES: Mayo endoscopic subscore; SCCAI: Simple Clinical Colitis Activity Index.

**Table 3:** Clinical performance characteristics of two consecutive fecal-calprotectin < 250 *μ*g/g as a surrogate marker of mucosal healing after treatment for active ulcerative colitis.

Sensitivity (95% CI) (%)	84.2 (60.4–96.6)
Specificity (95% CI) (%)	100 (2.5–100)
PPV (95% CI) (%)	100 (79.4–100)
NPV (95% CI) (%)	25.0 (0.6–80.6)

CI: confidence interval; PPV: positive predictive value; NPV: negative predictive value.
